# Application of a new sternoclavicular hook plate in bipolar clavicle injuries

**DOI:** 10.3389/fsurg.2022.935653

**Published:** 2023-01-10

**Authors:** Han Zhang, Zhong Zhu, Lie Lin, Guoping Cai

**Affiliations:** Department of Orthopedic, Taizhou Hospital of Zhejiang Province Affiliated to Wenzhou Medical University, Linhai, China

**Keywords:** sternoclavicular hook plate, shoulder locking hook plate, hook plate, clavicle bipolar injury, operation

## Abstract

**Objective:**

To evaluate the safety and effectiveness of using a new sternoclavicular hook plate combined with a shoulder locking hook plate for the treatment of bipolar clavicle injuries.

**Methods:**

Retrospective analysis of 7 patients with bipolar clavicle injury, all-male, with a mean age of 51.1 years, who underwent a new sternoclavicular hook plate combined with a shoulder clavicular hook plate implantation.

**Results:**

All 7 patients were fixed with a sternoclavicular hook plate combined with a repositioned shoulder locking hook plate and received 13–24 months of follow-up. There were no postoperative complications, no wound infections, and no plate or screw fractures. The mean ASES score was 94.3 ± 2.8.

**Conclusion:**

The safety and effectiveness of a new sternoclavicular hook plate combined with a shoulder locking hook plate in the treatment of bipolar clavicle injuries.

## Introduction

Bipolar injuries of the clavicle are reported very rarely and are often due to high-energy injuries (1–3). There are various types of clavicle bipolar injuries, including fractures of both ends of the clavicle, dislocation of both ends of the clavicle, fractures of the proximal clavicle with distal clavicle dislocation, and fractures of the distal clavicle with proximal clavicle dislocation (4). There is still controversy regarding the best treatment for bipolar clavicle fractures, with nonoperative treatment achieving good results in asymptomatic, elderly, or low-demand patients. However, nonoperative treatment may result in poor function, pain, and bone discontinuity, and long-term limb immobilization can severely compromise the quality of life. Therefore, surgical treatment is often used for younger patients, those with high needs, or those who do not tolerate long-term limb immobilization (5, 6). A variety of surgical techniques have been used to treat bipolar clavicle injuries, but there is still no standardized surgical procedure. In this study, we designed and used a new sternoclavicular hook plate in combination with a clavicular hook plate for the treatment of bipolar clavicle injuries.

## Methods

### General information

From September 2017 to January 2020, patients (range of ages 34–68) with bipolar clavicle injury were admitted to our department, and the surgical approach of sternoclavicular hook plate combined with shoulder locking hook plate was used. Inclusion criteria: [1] Age over 18 years. [2] Imaging confirmation of acute traumatic bipolar clavicle injury. [2] The surgical approach used a sternoclavicular hook plate combined with a shoulder locking hook plate. [3] With complete follow-up information. Exclusion criteria: [1] Underwent conservative or other treatment. [2] Inapprehensive follow-up information. Ultimately, seven patients, all of whom permitted informed consent, were enrolled in this study. The sternoclavicular hook plate and related auxiliary instruments (Patent No. ZL2003201079412 and ZL201220667654.6) were designed by ourselves and manufactured by Zhejiang Kangwell Medical Co., Ltd (China) for fixing proximal clavicle injuries. The Ethics Committee of Zhejiang Taizhou Hospital approved this study.

### Pre-operative evaluation

Offering no contraindications to surgery, all patients underwent a standard preoperative evaluation, including preoperative case, physical examination, pain score, chest CT, and electrocardiogram.

### Surgical procedure

**Figure 1:**
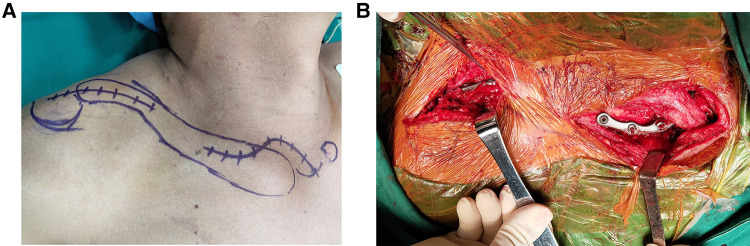
Plate fixation procedure in a patient with bipolar clavicle injury. (**A**) Schematic diagram of the plate position. (**B**) Both clavicle ends were repositioned using leverage, and then the new sternoclavicular and acromioclavicular plates were placed respectively.

All cases were handled by the same surgeon. Likewise, all patients were supine on the operating table with appropriate padding of the affected limb and general anesthesia was administered. A T-shaped incision of approximately 10 cm was made from the mid-clavicle to the sternal stalk, centered on the medial end of the clavicle, and the skin layers were incised and subperiosteal dissection was performed to expose the sternoclavicular joint, sternal stalk, and proximal clavicle. A transverse right lateral clavicle incision of approximately 8 cm in length was made, with the distal end of the incision curved posteriorly at the level of the acromioclavicular joint, and the skin layers were incised to the surface of the clavicle to expose the acromioclavicular joint, distal clavicle and acromion. In light of the rotation occurring in the clavicle as detected by preoperative CT, the clavicle ends on both sides were repositioned using leverage, and then the new sternoclavicular and acromioclavicular plates were placed respectively (Figure 1). Specifically, the new sternoclavicular hook plate was wound out from the upper edge of the sternal shank from the posterior side to the anterior orifice. In anterior dislocation, the clavicle was reset posteriorly by lever action, which also re-set the proximal fracture, followed by fixing the plate on the clavicle with screws to complete the fixation. In posterior dislocation, the plate was applied to reset the fracture dislocation by lifting action, with spacers and nuts at the threads of the hook head end to prevent the tendency of posterior dislocation (Supplementary Figure S1).

### Postoperative management

Simple exercises such as pendulum movements for the first 4 weeks. passive flexion and supination exercises after 4 weeks and return to daily activities and work 3 months after surgery. The plate is removed 6–12 months postoperatively according to the disappearance of the fracture line for for all cases.

### Follow-up

Intraoperatively and postoperatively we evaluated for early complications regarding nerve, vascular, and tracheal injuries, as well as performed x-rays or CT to assess the position of the internal fixation. Clinical and radiological evaluations were performed weekly for the first month of follow-up and then every month. The healing of the fracture or dislocation was measured by CT at 6 months postoperative period, followed by plate removal if the fracture was well-healed. During pre-surgery and the last follow-up, we assessed the patient's physical function and ability using the American Society of Shoulder and Elbow (ASES) scoring system, and the Quick Disabilities of the Arm, Shoulder and Hand questionnaire (QuickDASH), and the Constant score. Patients' postoperative pain levels were assessed using a visual analog score (VAS), with 0 indicating no pain and 10 indicating severe pain.

## Results

### Clinical characteristics of patients

The mean age of the seven patients was 51.1 years, and all were male patients. There were 2 fractures of both ends of the clavicle, 2 dislocations of both ends of the clavicle, 1 proximal clavicle fracture with distal clavicle dislocation, and 2 distal clavicle fractures with proximal clavicle dislocation. 2 had combined rib fractures, 1 had combined femur fractures, and 1 had combined head injury. The injury mechanisms were all high-energy injuries: car accident in 4 cases, fall from height in 1 case, fall on the motorcycle in 1 case, and slip and fall in 1 case. In this study, all patients were treated surgically, with a mean operative time of 3 h and a mean blood loss of 50 ml. All procedures were performed without intraoperative complications such as nerve, vascular, or tracheal injuries. Meanwhile, All surgeries had no postoperative wound infections and no complications such as joint re-dislocation.

### Imaging results

**Figure 2:**
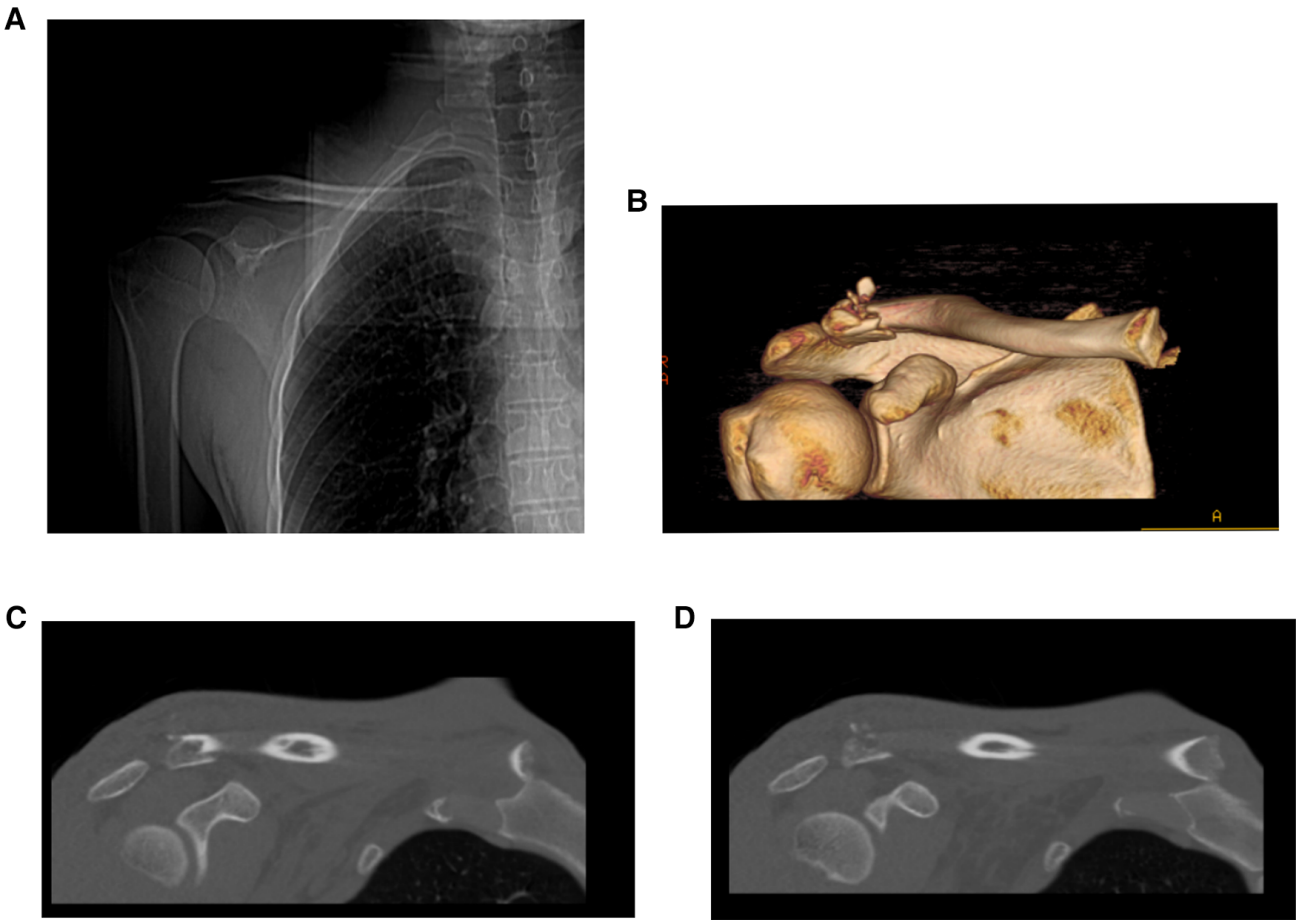
Imaging of a patient with a distal clavicle fracture with proximal clavicle dislocation. (**A**) X-ray images. (**B–D**) CT images.

**Figure 3:**
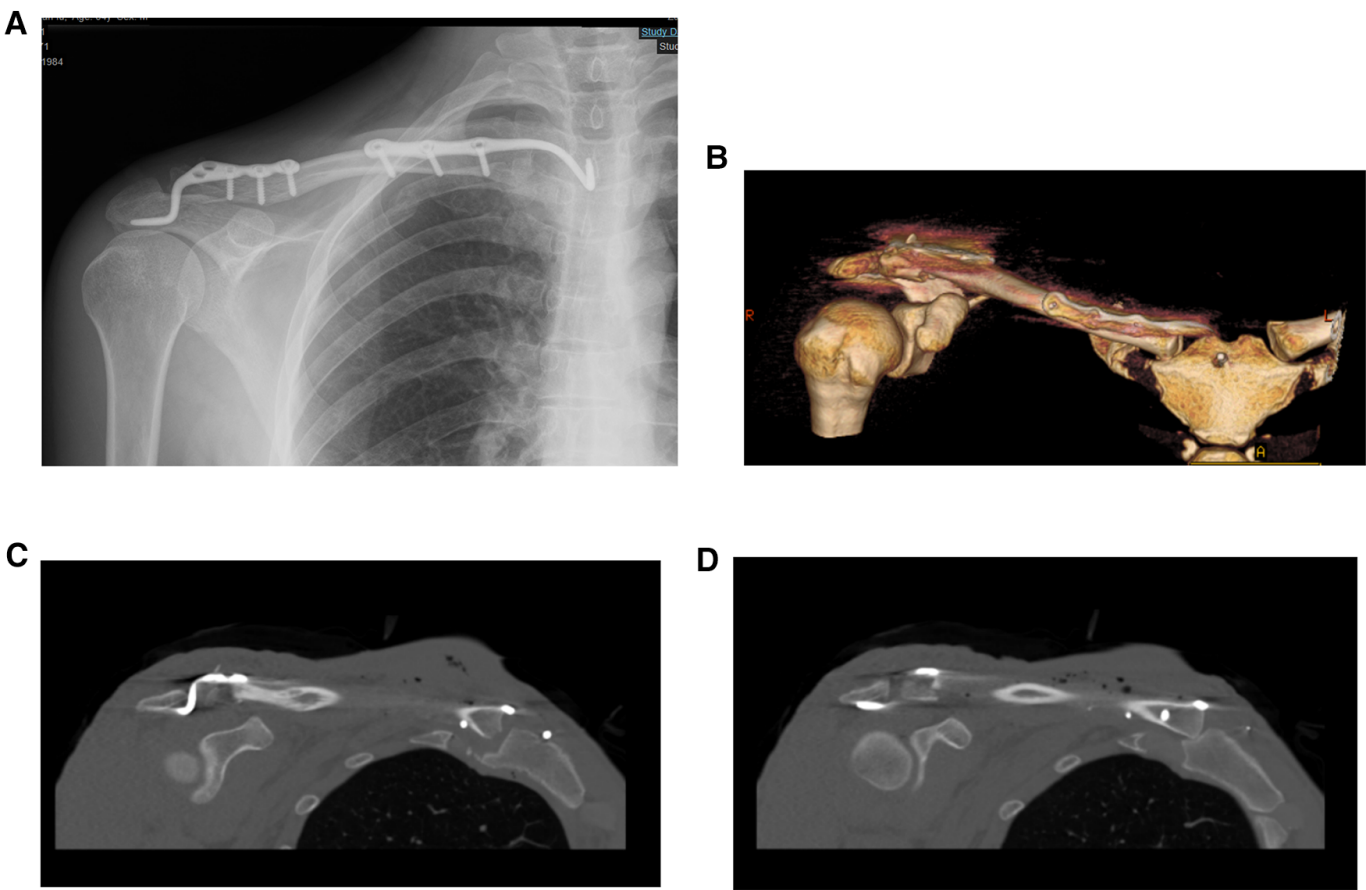
Postoperative imaging of the patient. (**A**) X-ray image. (**B–D**) CT images.

As compared to pre-operative (Figure 2), postoperative x-ray or CT showed good fracture or dislocation repositioning, and good position and angle of the sternoclavicular hook plate and shoulder locking hook plate. During the follow-up, no re-fracture or dislocation was found. CT showed significant bone scab formation on the sternal edge, but the sternoclavicular joint was well-matched (Figure 3).

### Functional results

**Figure 4:**
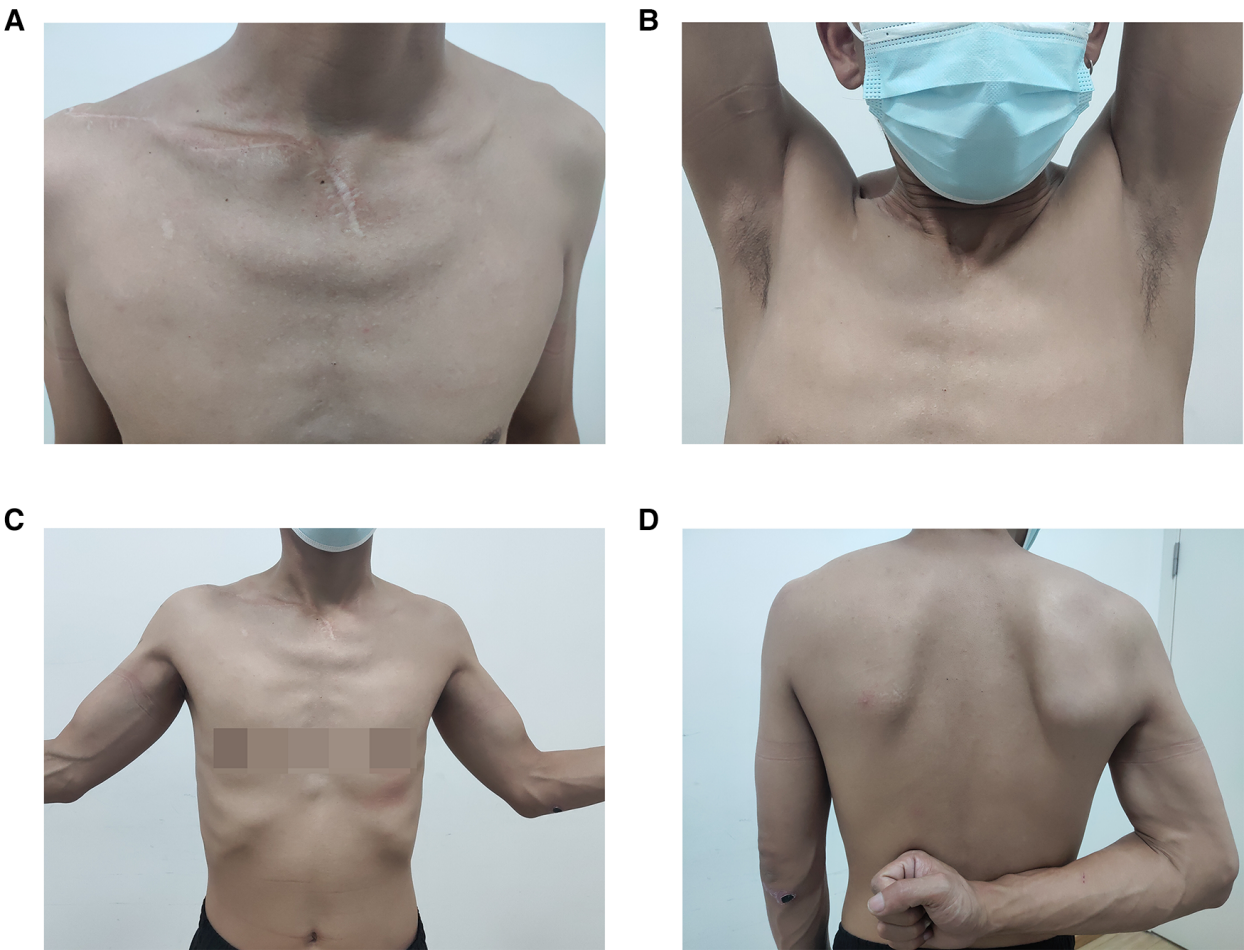
Functional recovery of the patient after surgery (**A–D**).

We performed 13–24 months postoperative follow-up (mean follow-up 18.2 months), in which the mean ASES score was 94.3 ± 2.8 (range: 90–98), the mean QuickDASH score was 3.9 ± 1.9 (range: 1.7–6.7), and the mean Constant score was 94.1 ± 3.1 (range 89–99) in all patients at the final follow-up (Table 1). All patients had good functional recovery of the shoulder joint at the final follow-up (Figure 4).

**Table 1:** Clinical features associated with 7 patients with bipolar clavicle injuries.

Patient No./Sex/Age	medial injury	lateral injury	ASES	QuickDASH	VAS	Costant Score
1/M/34y	fracture	fracture	98	1.7	0	99
2/M/56y	fracture	dislocation	92	5.8	1	94
3/M/68y	fracture	fracture	90	6.7	2	89
4/M/49y	anteriorly/dislocation	dislocation	96	2.5	0	95
5/M/40y	anteriorly/dislocation	fracture	96	3.3	0	94
6/M/51y	anteriorly/dislocation	dislocation	95	2.5	1	96
7/M/60y	posteriorly/dislocation	fracture	93	5	1	92

### Pain outcomes

All patients in this study recovered well from their pain scores and VAS recovered from 7.14 ± 0.69 (range: 6–8) before surgery to 0.71 ± 0.76 (range: 0–2) at the last follow-up.

## Discussion

Reports of bipolar clavicle injuries are rare, and there is still controversy regarding the best treatment for bipolar clavicle fractures (5, 7). Although good results have been reported for nonoperative treatment of bipolar clavicle injuries (3). However, most scholars recommend surgical treatment for young, active patients in whom anatomic repositioning cannot be obtained with nonoperative treatment and who have residual deformity, pain, or functional limitations (5), but a standardized surgical procedure has still not been reached.

The exact mechanism of clavicular bipolar injury remains uncertain. Some believe that multidirectional forces cause the clavicle to rotate around its center resulting in bipolar clavicle injury (8). This fits the situation in our case. We can see the rotation of the clavicle occurring on preoperative CT as well as intraoperatively. Synergistic resetting by leverage is required for the resetting. Some scholars believe that good stability can be provided by correcting the rotation of the clavicle and fixing the lateral end of the clavicle alone (3). The fixation of the medial end of the clavicle is still controversial (9). The sternoclavicular joint lacks bony stability and is one of the most unstable joints in the human body. Its stability is mainly maintained by soft tissues such as the joint capsule, anterior and posterior sternoclavicular ligaments, interclavicular ligaments, and costoclavicular ligaments (10). Moreover, the clavicle rotates when the bipolar clavicle is injured (11). Therefore, we choose to use the clavicle hook plate combined with the shoulder lock hook plate to treat the injury of the medial end of the clavicle. At present, most clinics apply the shoulder-clavicular hook plate inserted into the medullary cavity of the sternum or placed on the posterior side of the sternum (12), however, the unyielding nature of the plate makes the surgical operation more difficult and reduces the efficacy of joint repositioning. Therefore, our group developed a new type of sternoclavicular joint hook plate with the hooked end inserted into the medullary cavity of the sternum for fixation.

For the seven patients in this study, we used the shoulder lock hook plate combined with the new sternoclavicular hook plate to treat bipolar clavicle injuries, and the surgery proved to be safe and effective. Good functional recovery and significant pain relief were also evident in the associated functional and pain scores. The relevant imaging examinations showed that the repositioning angle and position were good and no re-fracture or dislocation occurred.

The new sternoclavicular hook plate is a specialized category of plate that we designed for the treatment of medial clavicle injuries. The advantages of this plate for the treatment of bipolar clavicle fractures through our treatment experience are as follows. As a first point, the mechanical stability was excellent. After sternoclavicular plate fixation, the sternoclavicular foramen serves as the axis of movement, which allows the patient to retain the micro-movement of the sternoclavicular joint and practice early functional exercises. Our functional scores suggested that the injured recovered well. Second, bipolar clavicle injury is usually accompanied by rotation of the clavicle. We adopted bilateral plate fixation, which could better utilize the leverage principle to reset the bipolar clavicle fracture and address the rotation of the clavicle simultaneously. However, we need to be extremely cautious about the intraoperative operation to avoid damage to the nerves, blood vessels or organs posterior to the bone. A nerve stripper should be applied to softly pluck the nerves and blood vessels behind the sternum before inserting the hook part of the sternoclavicular hook plate into the sternum, as well as a restrictor is required for drilling in the sternum. Furthermore, in the case of posterior dislocation of the sternoclavicular joint, thin spacers and locking nuts should be installed on the head of the sternoclavicular hook plate to avoid backward displacement. As a point of note, we observed that the activity of the upper extremity is transmitted through the clavicle to the hook plate, and the head of the hook is a major force release point that creates an axis of activity, sequentially resulting in bone resorption around the head of the hook on the sternal side. If strenuous activity is performed early after the procedure, the sternal bone is not sufficient to stop the tendency of clavicle dislocation, especially in osteoporotic conditions, which may lead to a cut and result in failure of internal fixation. Therefore we recommend simple exercises for the first 4 weeks after surgery to prevent damage to the sternum due to early strenuous activity and we propose that the plate should be removed.

Of course, due to the low incidence of bipolar clavicle injuries and our small number of cases, the combination of a shoulder-lock hook plate with a new sternoclavicular hook plate has not been studied in the treatment of more complex bipolar clavicle injuries. In addition, our design of this new hook plate is based on Chinese anatomical data at the sternoclavicular joint, which may limit its application in other races.

## Conclusion

In bipolar clavicle injuries case, for which anatomic repositioning cannot be obtained with nonoperative treatment and may interfere with functional recovery, we may opt for surgical treatment, and firm internal fixation may be more helpful for functional recovery of the patient. The shoulder locking hook plate combined with the new sternoclavicular hook plate has a good prospect of application in the treatment of bipolar clavicle injuries.

## Data Availability

The raw data supporting the conclusions of this article will be made available by the authors, without undue reservation.
