# Supplementary figures and images for: Application of a new sternoclavicular hook plate in bipolar clavicle injuries
Source: Front Surg. 2023 Jan 10;9:935653. doi: 10.3389/fsurg.2022.935653 (PMC9871760; doi:10.3389/fsurg.2022.935653)

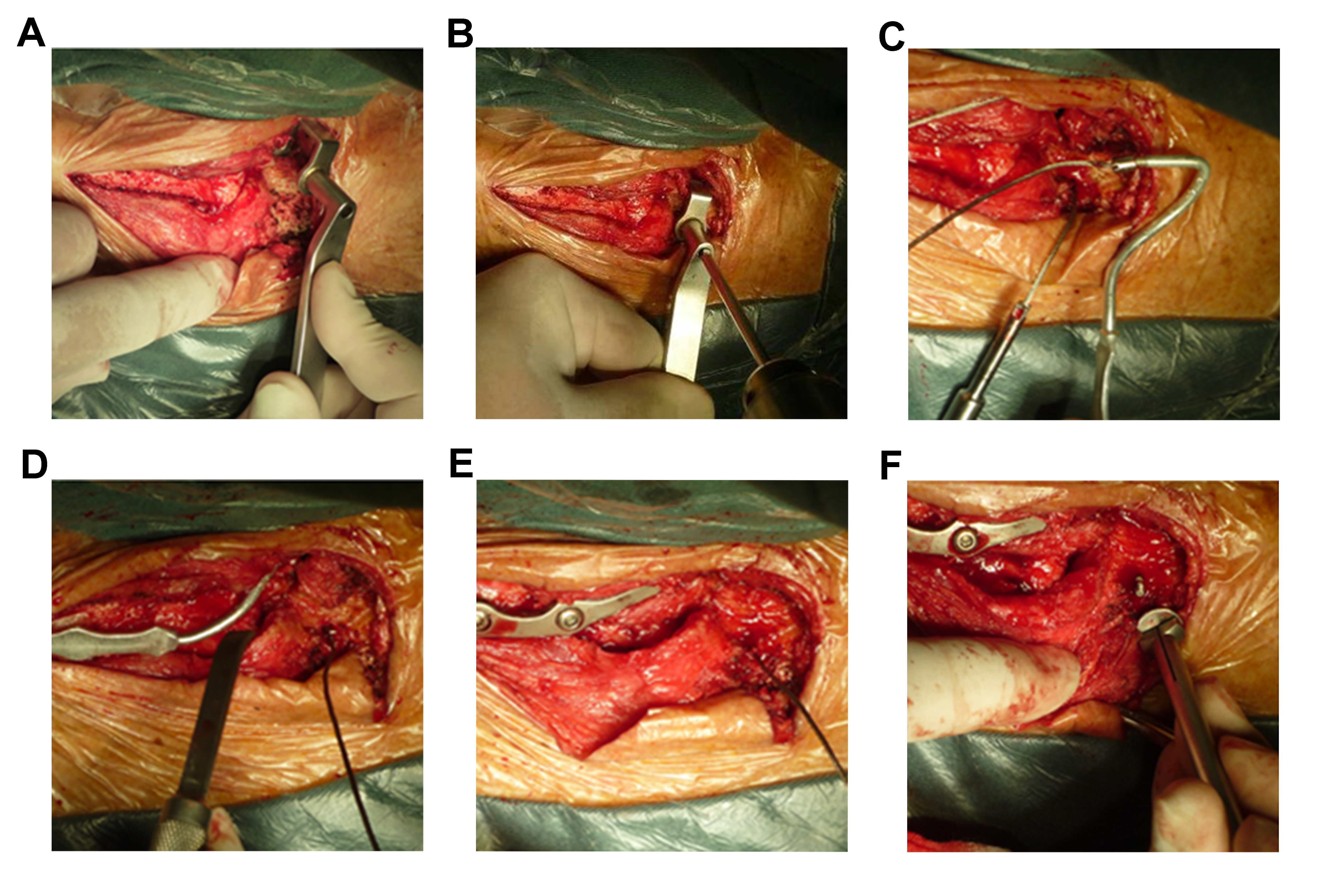

Supplement: Supplementary file 1 [file Image1.tif]
